# Adherence to a Plant-Based Diet and Consumption of Specific Plant Foods—Associations with 3-Year Weight-Loss Maintenance and Cardiometabolic Risk Factors: A Secondary Analysis of the PREVIEW Intervention Study

**DOI:** 10.3390/nu13113916

**Published:** 2021-11-01

**Authors:** Ruixin Zhu, Mikael Fogelholm, Sally D. Poppitt, Marta P. Silvestre, Grith Møller, Maija Huttunen-Lenz, Gareth Stratton, Jouko Sundvall, Laura Råman, Elli Jalo, Moira A. Taylor, Ian A. Macdonald, Svetoslav Handjiev, Teodora Handjieva-Darlenska, J. Alfredo Martinez, Roslyn Muirhead, Jennie Brand-Miller, Anne Raben

**Affiliations:** 1 Department of Nutrition, Exercise and Sports, Faculty of Science, University of Copenhagen, Rolighedsvej 30, Frederiksberg C, 1958 Copenhagen, Denmark; ruixinzhu@nexs.ku.dk (R.Z.); gmp@nexs.ku.dk (G.M.); 2 Department of Food and Nutrition, University of Helsinki, P.O. Box 66, 00014 Helsinki, Finland; mikael.fogelholm@helsinki.fi (M.F.); elli.jalo@helsinki.fi (E.J.); 3 Human Nutrition Unit, Department of Medicine, School of Biological Sciences, University of Auckland, Auckland 1042, New Zealand; s.poppitt@auckland.ac.nz (S.D.P.); m.silvestre@auckland.ac.nz (M.P.S.); 4 CINTESIS, NOVA Medical School, NMS, Universidade Nova de Lisboa, 1169-056 Lisboa, Portugal; 5 Institute for Nursing Science, University of Education Schwäbisch Gmünd, Oberbettringerstrasse 200, 73525 Schwäbisch Gmünd, Germany; maija.huttunen-lenz@ph-gmuend.de; 6 Applied Sports, Technology, Exercise and Medicine (A-STEM) Research Centre, Swansea University, Swansea SA1 8EN, UK; g.stratton@swansea.ac.uk; 7 Finnish Institute for Health and Welfare, P.O. Box 30, 00271 Helsinki, Finland; jouko.sundvall@outlook.com (J.S.); laura.raman@thl.fi (L.R.); 8 Division of Physiology, Pharmacology and Neuroscience, School of Life Sciences, Queen’s Medical Centre, Nottingham NG7 2UH, UK; moira.taylor@nottingham.ac.uk; 9 Division of Physiology, Pharmacology and Neuroscience, School of Life Sciences, Queen’s Medical Centre, MRC/ARUK Centre for Musculoskeletal Ageing Research, ARUK Centre for Sport, Exercise and Osteoarthritis, National Institute for Health Research (NIHR) Nottingham Biomedical Research Centre, Nottingham NG7 2RD, UK; ian.macdonald1@nottingham.ac.uk; 10 Department of Pharmacology and Toxicology, Medical University of Sofia, 1000 Sofia, Bulgaria; svhandjiev@gmail.com (S.H.); teodorah@abv.bg (T.H.-D.); 11 Department of Nutrition and Physiology, University of Navarra, 31008 Pamplona, Spain; jalfmtz@unav.es; 12 Precision Nutrition and Cardiometabolic Health Program, IMDEA-Food Institute (Madrid Institute for Advanced Studies), CEI UAM + CSIC, 28049 Madrid, Spain; 13 Centro de Investigacion Biomedica en Red Area de Fisiologia de la Obesidad y la Nutricion (CIBEROBN), 28029 Madrid, Spain; 14 IdisNA Instituto for Health Research, 31008 Pamplona, Spain; 15 Charles Perkins Centre, School of Life and Environmental Sciences, University of Sydney, Camperdown, Sydney 2006, Australia; roslyn.muirhead@sydney.edu.au (R.M.); jennie.brandmiller@sydney.edu.au (J.B.-M.); 16 Steno Diabetes Center Copenhagen, 2820 Gentofte, Denmark

**Keywords:** plant-based dietary patterns, grains, legumes, nuts, fruits, vegetables, obesity, cardiovascular disease

## Abstract

Plant-based diets are recommended by dietary guidelines. This secondary analysis aimed to assess longitudinal associations of an overall plant-based diet and specific plant foods with weight-loss maintenance and cardiometabolic risk factors. Longitudinal data on 710 participants (aged 26–70 years) with overweight or obesity and pre-diabetes from the 3-year weight-loss maintenance phase of the PREVIEW intervention were analyzed. Adherence to an overall plant-based diet was evaluated using a novel plant-based diet index, where all plant-based foods received positive scores and all animal-based foods received negative scores. After adjustment for potential confounders, linear mixed models with repeated measures showed that the plant-based diet index was inversely associated with weight regain, but not with cardiometabolic risk factors. Nut intake was inversely associated with regain of weight and fat mass and increments in total cholesterol and LDL cholesterol. Fruit intake was inversely associated with increments in diastolic blood pressure, total cholesterol, and LDL cholesterol. Vegetable intake was inversely associated with an increment in diastolic blood pressure and triglycerides and was positively associated with an increase in HDL cholesterol. All reported associations with cardiometabolic risk factors were independent of weight change. Long-term consumption of nuts, fruits, and vegetables may be beneficial for weight management and cardiometabolic health, whereas an overall plant-based diet may improve weight management only.

## 1. Introduction

Cardiovascular diseases (CVDs) have placed a substantial healthcare and economic burden on governments and individuals [1]. Obesity is a major risk factor for CVDs [1]. Plant-based diets (PBDs) recommended by European food-based dietary guidelines [2] and the EAT-Lancet Commission [3] may be beneficial in terms of environmental sustainability, particularly if plant-based proteins replace animal-based foods such as red meat [2]. PBDs may also assist in weight management and prevention of CVDs [4,5,6,7,8,9] and improve multiple cardiometabolic risk factors [5].

Many previous randomized controlled trials (RCTs) and observational studies have explored the association of a vegetarian or a PBD with weight loss (WL) [8] or weight gain [6] or BMI [5]. Some prospective cohort studies have explored the association of PBDs with risk of CVDs [10], whereas previous evidence on PBDs and cardiometabolic risk factors was mainly based on cross-sectional studies and small-scale, short- or medium-term RCTs [11,12,13,14,15,16,17]. Long-term data on adherence to a PBD and weight regain and cardiometabolic risk factors during weight-loss maintenance (WLM), particularly after diet-induced rapid WL, are largely lacking.

Specific components of a PBD may also have an important role to play in weight management and cardiometabolic health. Certain plant foods such as whole grains, vegetables, fruits, legumes, and nuts are rich in vitamins, minerals, antioxidants, unsaturated fatty acids, and dietary fiber [7]. These plant foods are considered healthy, with improved health outcomes [7]. Other plant foods such as sugar-sweetened beverages, cakes, and cookies have lower nutrient density and higher energy density [7]. These plant foods are regarded as unhealthy and may have negative effects on health [7]. To our knowledge, few long-term studies have to date explored consumption of plant foods and weight regain and cardiometabolic risk factors during WLM.

Therefore, the objective of the current study was to assess the longitudinal associations of adherence to an overall PBD and specific plant foods with WLM and cardiometabolic risk factors in adults with high risk of type 2 diabetes (T2D). Data from the PREVIEW study, a 3-year randomized trial aimed at examining the effects of diet and physical activity (PA) interventions on T2D prevention, were used. We hypothesized that consumption of an overall PBD and healthy plant foods would be inversely associated with weight regain and cardiometabolic risk factors.

## 2. Materials and Methods

### 2.1. Study Design

The PREVIEW study was a 3-year, large-scale, 2 × 2 factorial randomized trial. It was conducted at 8 study centers including Denmark, Finland, The Netherlands, the UK, Spain, Bulgaria, New Zealand, and Australia. The detailed information has been described elsewhere [18], and the main results have been published [19,20]. Briefly, the PREVIEW study was designed to examine the effect of a high protein-low glycemic index (GI) diet vs a moderate protein–moderate GI diet (25 E% protein and GI < 50 vs. 15 E% protein and GI > 56) combined with 2 PA programs (high intensity or moderate intensity) on T2D incidence in adults with overweight or obesity and pre-diabetes. The primary endpoint was T2D incidence. The participants underwent an 8-week weight loss (WL) period, and during this period, they were instructed to consume a low energy total meal replacement diet containing 3.4 MJ·day^−1^. After this period, participants started a 148-week WLM period and received 1 of the 4 diet–PA interventions. The intervention diets were consumed ad libitum during WLM, and the participants were given examples of eating plans, cooking books, and food-exchange lists. Both diet interventions included the recommendation of whole grain cereals. A behavioral modification program (PREMIT) and 17 group visits were conducted throughout the intervention to improve dietary and PA compliance [21]. Dietary compliance was evaluated using 4-day food records. In addition, urinary nitrogen or urea analyses were done on 24 h urine samples to assess compliance to the diets, i.e., protein intake. PA compliance was evaluated using 7-day accelerometry.

The PREVIEW study was designed and conducted in line with the Declaration of Helsinki and its latest amendments. The protocol of the PREVIEW study was reviewed and approved by the following Human Ethics Committees at each intervention center. Denmark: The Research Ethics Committees of the Capital Region, ethical approval code: H-1-2013-052; Finland: Coordinating Ethical Committee of HUS (Helsinki and Uusimaa Hospital District), ethical approval code: HUS/1733/2017; the UK: UK National Research Ethics Service (NRES) and East Midlands (Leicester) Ethics Committee, ethical approval code: 13/EM/0259; the Netherlands: Medical Ethics Committee of the Maastricht University Medical Centre, ethical approval code: NL43054.068.13/METC 13-3-008; Spain: Research Ethics Committee of the University of Navarra, ethical approval code: 71/2013; Bulgaria: Commission on Ethics in Scientific Research with the Medical University-Sofia (KENIMUS), ethical approval code: 4303/13.06.2014; Australia: The University of Sydney, Human Research Ethics Committee (HREC), ethical approval code: 2013/535; and New Zealand: Health and Disability Ethics Committees (HDEC), ethical approval code: X14-0408.

The current analysis was an exploratory analysis based on the secondary outcomes of the PREVIEW study. Given that only 5 study centers (Finland, the UK, Bulgaria, New Zealand, and Australia) provided food intake data in g·day^−1^ or serving size·day^−1^ and full plant food categories, only data from the WLM phase (8–156 weeks) from participants at these 5 study centers were included.

### 2.2. Study Population

Participants aged 25–70 years with overweight (BMI 25–29.9 kg·m^−2^) or obesity (BMI ≥ 30 kg·m^−2^) and pre-diabetes were recruited between June 2013 and April 2015. Pre-diabetes was defined as impaired FPG (FPG of 5.6–6.9 mmol·L^−1^) or impaired glucose tolerance (2-h plasma glucose of 7.8–11.0 mmol·L^−1^ and FPG < 7.0 mmol·L^−^^1^) after an oral glucose tolerance test (oral ingestion of 75 g of glucose) [22]. Participants who were diagnosed with diabetes (T2D or type 1 diabetes) prior to the study or who were non-compliant with the intervention were excluded. Eligible participants were enrolled, underwent randomization by gender and age, and started WL. Participants who lost ≥8% of initial BW after the WL phase were eligible to continue, entering the 148-week WLM period. In the current analysis, we included those with available plant food data (and full plant food categories) at 26 weeks and plausible energy intake (2520–14700 kJ·day^−1^ for women and 3360–17640 kJ·day^−1^ for men) [23]. All participants provided written informed consent.

### 2.3. Assessment of Dietary Intake and Adherence to an Overall Plant-Based Diet

Dietary intake was estimated using self-administered 4-day food records on 4 consecutive days, including 3 weekdays and 1 weekend day. The 4-day food records were collected at 26, 52, 104, and 156 weeks. Participants were encouraged to record their diet by weighing foods and drinks using a weigh scale or household measures in the absence of a scale. Standard household measures such as cup, spoon, glass, and portions were explained. Additionally, participants were asked to describe the food in detail (e.g., type of foods, cooking methods, and ingredients). Food records were entered into the national nutrient analysis software, i.e., AivoDiet (Finland), Nutritics (the UK), Nutrition Calculation (Bulgaria), and Foodworks (Australia and New Zealand). Food intake at each time point was calculated as the average of 4 days and expressed in g·day^−1^ or serving·day^−1^. Serving sizes were converted to grams of food [23].

We created 11 food groups according to nutrients and culinary similarities within the larger categories of plant foods and animal-based foods (Table 1). Plant food groups included total grains and potatoes, legumes, nuts, vegetables, and fruits. Animal-based food groups included dairy products, eggs, red meat, processed meat, poultry and fish/seafood. Adherence to an overall PBD was evaluated using a plant-based diet index (PDI), modified from Satija et al. [24]. As conversion from serving·day^−1^ to g·day^−1^ of high-sugar products may introduce bias, they were not included in the PDI. In addition, in our dietary data, there was not a specific potato group. Potatoes and potato products were considered as 1 group. For the PDI calculation, food groups were divided into quintiles of consumption (g·day^−1^) and given positive (between 1 and 5) or negative (between –5 and –1) scores. For positive scores, participants in the highest quintile were assigned a score of 5 and those in the lowest quintile were assigned a score of 1. All plant food groups were given positive scores and all animal food groups were given negative scores. Scores based on the 11 food groups were summed to obtain the index. Higher PDI reflected lower consumption of animal-based foods.

### 2.4. Assessment of Outcomes

Outcomes including body weight (BW), fat mass (FM), waist circumference (WC), fasting plasma glucose (FPG), fasting insulin, glycosylated hemoglobin A_1c_ (HbA_1c_), homeostatic model assessment of insulin resistance (HOMA-IR), fasting triglycerides, total cholesterol, high-density lipoprotein cholesterol (HDL cholesterol), low-density lipoprotein cholesterol (LDL cholesterol), systolic blood pressure (SBP), and diastolic blood pressure (DBP) were measured at 8, 26, 52, 104, and 156 weeks. BW was measured in the fasting state (>10 h), with participants wearing light clothing or underwear. FM was determined by dual energy X-ray absorptiometry in the UK, Australia, and New Zealand and by bioelectrical impedance in Finland and Bulgaria. Blood samples were drawn from fasting participants’ antecubital veins. FPG, HbA_1c_, fasting insulin, fasting triglycerides, total cholesterol, HDL cholesterol, and LDL cholesterol were determined at the central laboratory of the Finnish Institute for Health and Welfare, Helsinki, Finland. HOMA-IR was calculated with the formula: fasting insulin (mU·L^−1^) × FPG (mmol·L^−1^)/22.5 [25]. SBP and DBP were determined using a validated automatic device on participants’ right arm after 5–10 min in a resting position.

### 2.5. Assessment of Covariates

Self-reported questionnaires were used to collect sociodemographic information including age, sex, ethnicity, and smoking status at baseline (0 weeks). PA was determined using 7-day accelerometry (ActiSleep+, ActiGraph LLC, Pensacola, FL, USA) and was expressed as counts·min^−1^, i.e., mean activity counts during valid wear time.

### 2.6. Statistical Analysis

For descriptive statistics, the normality of continuous variables was assessed by p–p plots and histograms. Approximately normally distributed variables are presented as means ± standard deviation (SD) and non-normal variables as medians (25th, 75th percentiles). Categorical variables are presented as absolute values and frequencies.

We conducted an available-case analysis and merged all participants into 1 group to assess longitudinal associations of adherence to an overall PBD (evaluated by PDI) and plant food intake with yearly changes in outcomes including BW and cardiometabolic risk factors, using adjusted linear mixed models with repeated measures. Model 1 was adjusted for fixed factors including age (continuous), sex (categorical; women and men), ethnicity (categorical; Caucasian, Asian, Black, Arabic, or other), intervention group (categorical), BMI at 8 weeks, weight or cardiometabolic risk factors at 8 weeks (continuous), and time (categorical) and random factors including study center (categorical) and participant-ID. For adherence to a PBD, model 2 was adjusted for covariates in model 1 plus fixed factors including time-varying PA (continuous), energy intake (kJ·day^−1^; continuous), and alcohol intake (g·day^−1^; continuous). For specific plant food intake, model 2 was additionally adjusted for consumption of animal-based foods (g·day^−1^; continuous) and other plant foods (g·day^−1^; continuous) as fixed factors. As dietary sodium intake may be associated with blood pressure [26], model 2 was additionally adjusted for sodium intake (g·day^−1^; continuous) when DBP or SBP was added as a dependent variable. Model 3 was adjusted for covariates in model 2 plus time-varying yearly weight change (continuous) as a fixed factor. Yearly changes were obtained by dividing changes in outcomes from 8 to 26, 52, 104, and 156 weeks by changes in years. To best represent the long-term dietary and PA patterns of participants during WLM, a cumulative average method [24,27] based on all available measurements of self-reported diet and device-measured PA was used. In this calculation, the 26-week diet was related to yearly changes in weight and cardiometabolic risk factors from 8 to 26 weeks; the average of the 26- and 52-week diets was related to yearly changes in weight and cardiometabolic risk factors from 8 to 52 weeks; the average of the 26-, 52-, and 104-week diets was related to yearly changes in weight and cardiometabolic risk factors from 8 to 104 weeks; the average of the 26-, 52-,104-, and 156-week diets was related to yearly changes in weight and cardiometabolic risk factors from 8 to 156 weeks. Cumulative average PA was calculated using the same method. Detailed information is included in Supplementary Materials Table S1. A sensitivity analysis was conducted by adding smoking status (categorical; daily, less than weekly, or no smoking) at 0 weeks in the abovementioned models. As the results were similar, they are not shown.

The associations of adherence to PBD with outcomes of interest are expressed as changes in outcomes per year induced by each 1 SD increment in PDI. To clarify the associations of PDI with BW, we also divided the participants into 2 groups, i.e., higher (n = 344 at 8 weeks) or lower (n = 344 at 8 weeks) adherence to PBD, at each time point separately according to PDI. We examined the difference in change in BW between the 2 groups using linear mixed models adjusted for the covariates in model 2. As the 2 groups were defined afresh at each time point, we compared the 2 groups at each time point regardless of the significance of time and group interaction. The associations of plant foods with outcomes of interest are expressed as changes in outcomes per year associated with each 1-serving size increment in plant food intake. The serving sizes were defined as follows: 75 g·day^−1^ for total grains and potatoes, 10 g·day^−1^ for legumes, 5 g·day^−1^ for nuts, 100 g·day^−1^ for vegetables, 50 g·day^−1^ for fruits, and 150 g·day^−1^ for combined consumption of vegetables and fruit—all based on medians and 25th and 75th percentiles of plant food intakes from dietary records over 3 years.

Missing data were not handled with multiple imputation because this method did not increase precision [28,29]. Data were analyzed using IBM SPSS v26.0 (Chicago, IL, USA). All *P* values were based on 2-sided tests and *P* < 0.05 was considered significant.

## 3. Results

**Figure 1:**
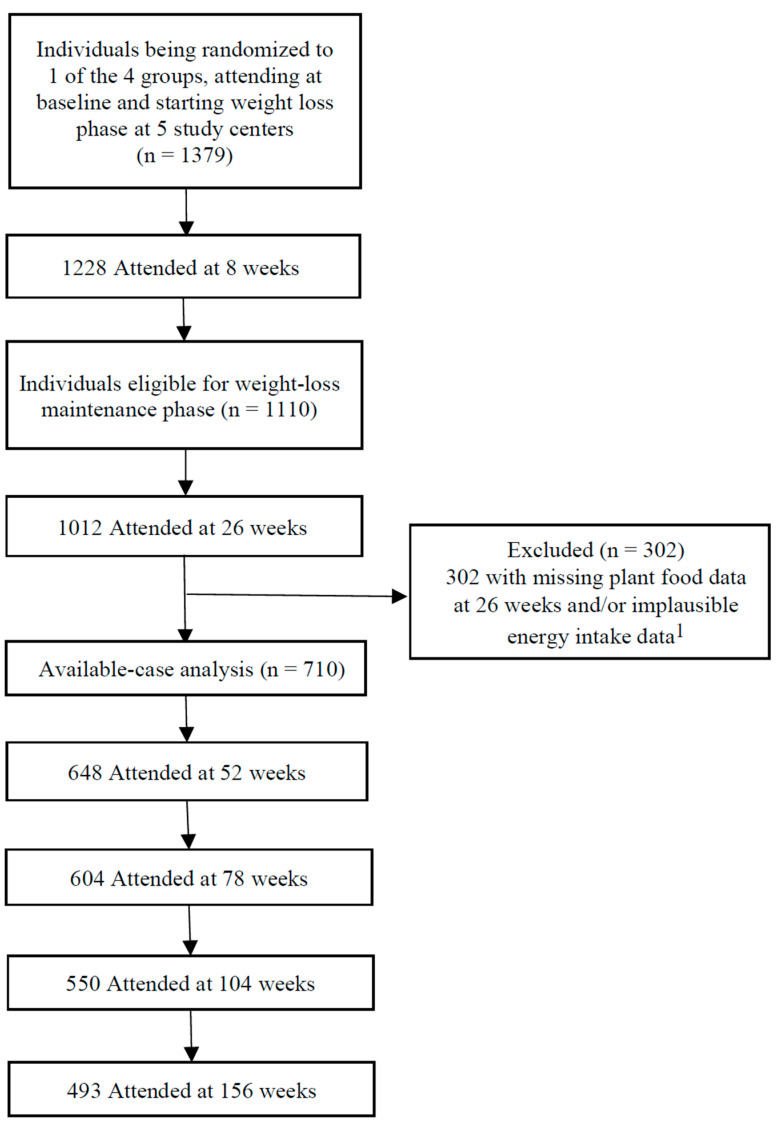
Participant flow diagram. ^1^ Individuals with missing plant food data at 26 weeks and/or implausible energy intake data (<2520 or >14,700 kJ·day^−1^ for women and <3360 or >17,640 kJ·day^−1^ for men) were excluded.

In the present available-case analysis, we included 710 participants and 2144–2336 observations of outcomes from available plant food data and full plant food categories at 26 weeks and plausible energy intakes (Figure 1). Of these, 493 participants completed the study. Participants who withdrew during WLM did so for personal reasons, including time constraints, moving away, and illness. The median age of participants (69% women) at the beginning of WLM was 57 years (range: 26–70 years) (Table 2). As there were no poultry and processed meat data from participants in Bulgaria, PDI analysis was based on 688 participants with both complete plant food and animal food data.

**Figure 2:**
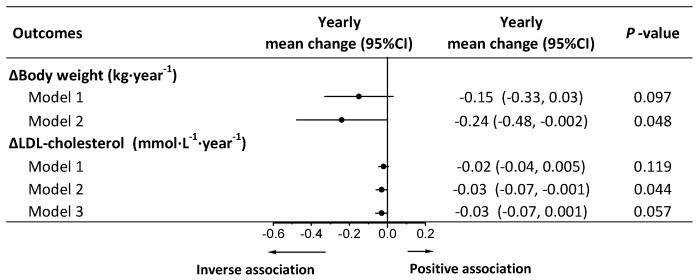
Longitudinal associations of adherence to a plant-based diet with yearly weight regain and changes in cardiometabolic risk factors during weight-loss maintenance. Yearly mean change and 95% CI of main effects indicating changes in body weight or cardiometabolic risk factors per year associated with a 1 standard deviation increment in plant-based diet index. Analyses were conducted using a linear mixed model with repeated measures. Model 1 was adjusted for fixed factors including age, sex, ethnicity, BMI at 8 weeks, body weight, or cardiometabolic risk factors at 8 weeks, and time and random factors including study center and participant ID. Model 2 was adjusted for covariates in model 1 plus fixed factors including time-varying physical activity, energy intake (kJ·day^−1^), alcohol intake (g·day^−1^). Model 3 was adjusted for covariates in model 2 plus time-varying yearly changes in body weight as a fixed factor. LDL cholesterol, low-density lipoprotein cholesterol.

Adherence to an overall PBD was inversely associated with weight regain and increment in LDL cholesterol in model 2, whereas after adjustment for weight change, the association of PBD with LDL cholesterol was lost (Figure 2). No associations were observed between PBD and other cardiometabolic risk factors in models 2 and 3 (Table S2). In all participants (n = 688), compared with those with lower adherence to PBD, participants with higher adherence to PBD had less weight regain at 52 and 104 weeks (Figure S1A). In completers (n = 493), compared with those with lower adherence to PBD, participants with higher adherence to PBD had less weight regain at 26, 52, and 104 weeks (Figure S1B).

**Figure 3:**
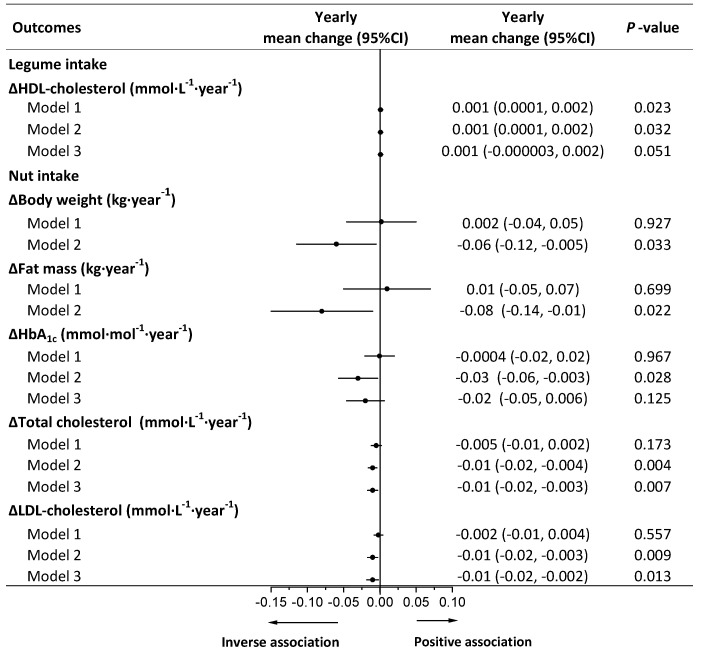
Longitudinal associations of legume (10 g·day^−1^) or nut (5 g·day^−1^) intake with yearly weight regain and changes in cardiometabolic risk factors during weight-loss maintenance. Yearly mean change and 95% CI of main effects indicating changes in body weight or cardiometabolic risk factors per year associated with 10 g increment in legume intake or 5 g increment in vegetable intake. Analyses were conducted using a linear mixed model with repeated measures. Model 1 was adjusted for fixed factors including age, sex, ethnicity, BMI at 8 weeks, body weight, or cardiometabolic risk factors at 8 weeks and time and random factors including study center and participant ID. Model 2 was adjusted for covariates in model 1 plus fixed factors including time-varying physical activity, energy intake (kJ·day^−1^), alcohol intake (g·day^−1^), animal-based food intake (g·day^−1^), and other plant food intake (g·day^−1^). Model 3 was adjusted for covariates in model 2 plus time-varying yearly changes in body weight as a fixed factor. HbA_1c_, glycosylated hemoglobin A_1c_; HDL cholesterol, high-density lipoprotein cholesterol; LDL cholesterol, low-density lipoprotein cholesterol.

Total grains and potatoes were not associated with any health outcomes in models 2 and 3 (Table S3). Legumes were positively associated with an increase in HDL cholesterol, whereas after adjustment for weight change, the association was lost (Figure 3). No associations were observed between legumes and weight regain or other cardiometabolic risk factors in models 2 and 3 (Table S4). Nuts were inversely associated with increments in BW, FM, HbA_1c_, total cholesterol, and LDL cholesterol in model 2. After adjustment for weight change, the association of nuts with HbA_1c_ was lost (Figure 3). No associations were observed between nuts and other cardiometabolic risk factors in models 2 and 3 (Table S5).

**Figure 4:**
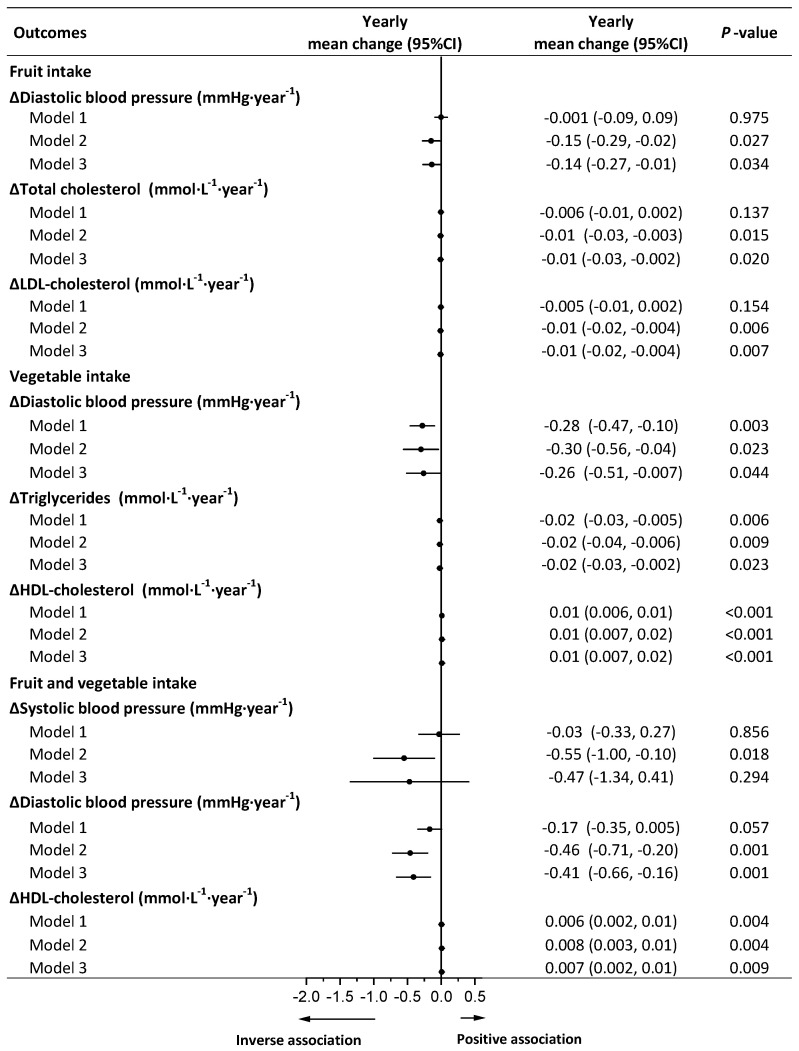
Longitudinal associations of fruit (50 g·day^−1^) or vegetable (100 g·day^−1^) or fruit and vegetable intake (150 g·day^−1^) with yearly changes in cardiometabolic risk factors during weight-loss maintenance. Yearly mean change and 95% CI of main effects indicating changes in cardiometabolic risk factors per year associated with 50 g increment in fruit intake or 100 g increment in vegetable intake or 150 g increment in fruit and vegetable intake. Analyses were conducted using a linear mixed model with repeated measures. Model 1 was adjusted for fixed factors including age, sex, ethnicity, BMI at 8 weeks, cardiometabolic risk factors at 8 weeks, and time and random factors including study center and participant ID. Model 2 was adjusted for covariates in model 1 plus fixed factors including time-varying physical activity, energy intake (kJ·day^−1^), alcohol intake (g·day^−1^), animal-based food intake (g·day^−1^), and other plant food intake (g·day^−1^); for systolic blood pressure and diastolic blood pressure, model 2 was additionally adjusted for dietary sodium intake (g·day^−1^). Model 3 was adjusted for covariates in model 2 plus time-varying yearly changes in body weight as a fixed factor. HDL cholesterol, high-density lipoprotein cholesterol; LDL cholesterol, low-density lipoprotein cholesterol.

In the available analysis, fruits were inversely associated with increments in DBP, total cholesterol, and LDL cholesterol after adjustment for weight change in model 3 (Figure 4). No associations were observed between fruits and weight regain or other cardiometabolic risk factors in models 2 and 3 (Table S6). Vegetables were inversely associated with DBP and triglycerides and were positively associated with HDL cholesterol, independent of weight change in model 3 (Figure 4). No associations were observed between vegetables and weight regain or other cardiometabolic risk factors in models 2 and 3 (Table S7). Combined vegetable and fruit intake was inversely associated with an increment in SBP, and DBP and was positively associated with an increase in HDL cholesterol in model 2, whereas after adjustment for weight change, only the associations of DBP and HDL cholesterol with combined vegetable and fruit intake remained significant in model 3 (Figure 4 and Table S8).

## 4. Discussion

In this 3-year, multi-center study, we examined the longitudinal associations of an overall PBD and specific plant foods with WLM and cardiometabolic risk factors in individuals with a high risk of developing T2D. We found that adherence to an overall PBD diet improved weight management. Consumption of nuts, fruits, and vegetables and fruits and vegetables was inversely associated with weight regain or cardiometabolic risk factors. Importantly, the reported associations with cardiometabolic risk factors were independent of weight change.

In the present analysis, we found that an overall PBD was inversely associated with weight regain, which is in agreement with findings from meta-analyses of RCTs [5,8,30] and prospective studies. Satija et al. [31] and Choi et al. [6] found inverse associations between adherence to a PBD and long-term weight gain. We did not find any associations between an overall PBD and cardiometabolic risk factors including glycemic markers, lipids, and blood pressure, after adjustment for weight change. On the contrary, Satija et al. [24,27,31] and Chen et al. [32] showed that an overall PDI was associated with smaller weight change or lower risk of T2D and coronary heart disease in three US prospective cohort studies. In addition, Glenn et al. [33] found that a plant-based Portfolio Diet was associated with a reduced CVD risk in the Women’s Health Initiative Prospective Cohort.

The mixed findings may be partly explained by differences in assessment of adherence to a PBD. In observational studies, compliance to a PBD was commonly assessed by dietary indices such as PDI (including 18 food groups) [24,27] and A Priori Diet Quality Score (including 46 food groups) [6]. Specifically, the PDI created by Satija et al. [27] included vegetable oils, tea, and coffee (as healthy plant foods) and sugar-sweetened beverages and sweets and desserts (as unhealthy plant foods). However, in our PDI version, we did not include these food groups. In addition, some prospective studies showed that compared with overall PDI, healthy PDI emphasizing whole grains, fruits, vegetables, nuts, legumes, and vegetable oils showed stronger associations with smaller weight change and lower risk of T2D and coronary heart disease [24,27,31]. Furthermore, we did not calculate healthy PDI by giving positive scores to whole grains, vegetable oils, and tea and coffee and giving negative scores to refined grains, sugar sweetened beverages, and sweets and desserts. Compared with healthy PBDs or vegetarian diets in the abovementioned studies, our PDI captured a “less healthy” diet. PBDs rich in healthy components have higher dietary fiber and micronutrients as well as lower energy, GI, and glycemic load [27]. In a previous secondary analysis of the PREVIEW study, we found positive associations between GI, glycemic load, and weight regain during WLM [34].

In terms of specific components of PBDs, we found that nuts were linked with improved weight management and cardiometabolic risk factors during WLM. Similar to our findings, meta-analyses of prospective studies and RCTs also showed that nuts improved weight management [35,36,37] and cardiometabolic risk factors including HOMA-IR and fasting insulin [38]. In a study based on a cross-sectional nutrition survey, compared with non-nut consumers, nut consumers had lower BW, BMI, and WC, whereas there were no differences in cardiometabolic risk factors including blood pressure, total cholesterol, HDL cholesterol, and HbA_1c_ [13]. The results, however, were limited by cross-sectional design, and causal inferences could not be drawn. Our study stands out because it used long-term, repeatedly measured, and updated dietary records and health outcomes, which not only provided a large number of observations, but also deeper insights into the causally relevant associations compared with cross-sectional studies and prospective studies with diet measured at baseline only.

We also observed that fruits, vegetables, and combined consumption of vegetables and fruits were associated with improved cardiometabolic risk factors. Similarly, in a population-based cross-sectional study, Mirmiran et al. [39] showed that consumption of fruits and vegetables was associated with lower concentrations of total cholesterol and LDL cholesterol. However, in a 12-week RCT, McEvoy et al. [40] did not find dose–response effects of fruit and vegetable intake on cardiometabolic risk factors including ambulatory blood pressure and plasma lipids. For grains, several RCTs and observational studies suggested that whole grains showed inverse effects on cardiometabolic risk factors [41,42,43], whereas refined grains showed positive effects [44,45]. However, there are conflicting reports of the associations of whole grains with obesity measures [46,47]. In the current study, total grains were not associated with any favorable change in health outcome. This may be because the grain group in the PREVIEW dietary dataset was not strictly based on whole grains, although whole grains were recommended to participants in each intervention arms during WLM.

The strengths of the current study are that this is the first multi-center, international study to explore associations of consumption of a PBD and specific plant foods with weight gain and cardiometabolic risk factors during 3-year WLM after diet-induced WL. Additionally, our study included individuals who met the pre-diabetes criteria (not just overweight/obesity) at the start. Furthermore, long-term, repeated dietary records were collected, which provided a large number of observations and a sufficient statistical power to adjust for important and multiple confounding factors including animal-based food intake and PA measured by accelerometry.

The current study also has some limitations. First, the attrition rate, especially at the end of the study, was higher than expected, which may affect the generalization of our findings. Second, the dietary data were obtained via 4-day food records, and misreporting, overreporting, or underreporting were inevitable [48]. Third, specific food groups such as refined grains, whole grains, and sugar sweetened beverages were not included in PDI calculation, which made this index less accurate when describing adherence to an overall PBD. It was not possible for us to investigate unhealthy PDI or specific unhealthy plant foods because these foods were specifically excluded in the dietary instructions. Furthermore, in order to make the results easy to understand, we divided the participants into higher or lower plant-based diet adherence groups according to PDI at each time point. Unlike RCTs, however, in the present analysis the participants were not randomly allocated to one of the two groups, which means that their baseline characteristics may be unbalanced. The statistical phenomenon “regression toward the mean” may have affected the two groups differently, making the natural variation in BW appear as true change [49]. Finally, residual and unmeasured confounders are possible in any observational analysis, which may create bias. Hyperuricemia, an independent risk factor for major CVD [50] and an important confounding factor, was not included in the current analysis. As we collected smoking status at baseline only (not at each time point), it was not possible to adjust for smoking status as a time-varying variable in the models. Adjustment for baseline values only may introduce bias because smokers may quit smoking during a long-term healthy lifestyle intervention.

## 5. Conclusions

This secondary analysis showed that long-term consumption of specific healthy plant foods including nuts, fruits, and vegetables improved weight management and cardiometabolic health, whereas adherence to an overall PBD benefited weight management only. Our findings support the hypothesis that specific components in a PBD are important as well. Although healthy and high-quality plant foods are currently recommended to individuals for reducing weight regain and the risk of developing CVDs, the observational nature of our analysis cannot establish a cause-and-effect relationship. The findings should be treated with caution because misreporting of food intake and unmeasured confounders are common.

## Figures and Tables

**Table 1:** Examples of food items in the 11 food groups.

Food Groups	Foods	Plant-Based Diet Index
**Plant**		
Grains	Bread rolls/baps/bagels, breads, cereal bars, cereal products, cereals, crackers/crispbreads, flours, grains, pastas, pastries/buns, rice, potatoes, potato products, pastry, plain cake, biscuits, and starch-based carbohydrate-rich snacks	Positive scores
Legumes	Pulses, beans, peas, lentils, and soy foods	Positive scores
Nuts	Nuts and seeds	Positive scores
Vegetables	leafy vegetables, dried vegetables, mushrooms, pickles/chutney, roots/tubers/bulbs, sea vegetables/algae, vegetable dishes, and avocado	Positive scores
Fruits	Fruits, canned fruit, and dried fruit	Positive scores
**Animal**		
Dairy	Milk products, cow’s milk, cream, creams, drinking yogurts, milkshakes/smoothies, processed milk/powders, yogurts, chilled desserts, and cheeses	Negative scores
Eggs	Eggs, egg products, and egg dishes	Negative scores
Red meat	Beef, pork, lamb, organ meats, meat dishes, and meat products	Negative scores
Processed meat	Frankfurters, bacon, corned beef, sausage, cured ham, and luncheon meat made from beef, pork, and poultry	Negative scores
Poultry	Poultry and poultry products, chicken, turkey	Negative scores
Fish/seafood	Fatty fish, fish products, low-fat fish, canned fatty fish, canned low-fat fish, seafoods, and crustaceans, including lobster and shrimps	Negative scores

**Table 2:** Characteristics of participants at baseline (0 weeks), the start of weight-loss maintenance (8 weeks), or 26 weeks.

Characteristics	
n	710
**Socio-demographics ^1^**	
Female, n (%)	491 (69.2)
Age (years)	57 (46, 63)
Height (m)	1.66 (1.61, 1.73)
Ethnicity, n (%)	
Caucasian	617 (86.9)
Asian	24 (3.4)
Black	19 (2.7)
Arabic	4 (0.6)
Other	46 (6.5)
Smoking, n (%)	
No	659 (92.8)
Yes, but less than weekly	17 (2.4)
Yes, at least daily	30 (4.2)
Missing	4 (0.6)
**Anthropometric outcomes and body composition ^2^**	
Body weight (kg)	86.1 ± 16.6
BMI (kg·m^−2^)	29.5 (26.7, 33.5)
Fat mass (kg)	33.5 ± 12.3
Waist circumference (cm)	100.2 ± 12.6
**Cardiometabolic risk factors ^2^**	
Fasting plasma glucose (mmol·L^−1^)	5.7 ± 0.6
HbA_1c_ (mmol·mol^−1^)	35.0 ± 3.1
HbA_1c_ (%)	5.4 ± 0.3
Fasting insulin (mU·L^−1^)	7.3 (5.3, 9.9)
HOMA-IR	1.8 (1.3, 2.5)
Systolic blood pressure (mmHg)	121.6 ± 15.8
Diastolic blood pressure (mmHg)	72.1 ± 9.5
Triglycerides (mmol·L^−1^)	1.0 (0.8, 1.2)
Total cholesterol (mmol·L^−1^)	4.1 ± 0.9
HDL cholesterol (mmol·L^−1^)	1.1 ± 0.2
LDL cholesterol (mmol·L^−1^)	2.4 (1.9, 3.0)
**Energy and food intake ^3^**	
Energy (kJ·day^−1^)	29,491 ± 7731
Grains (g·day^−1^)	206.1 (144.7, 276.6)
Legumes (g·day^−1^)	0.2 (0, 27.5)
Nuts (g·day^−1^)	3.2 (0, 10.8)
Fruits (g·day^−1^)	169.5 (83.8, 260.5)
Vegetables (g·day^−1^)	185.9 (97.1, 310.7)
Dairy (g·day^−1^)	361.0 (226.4, 501.0)
Eggs (g·day^−1^)	20.8 (5.1, 41.3)
Red meat (g·day^−1^)	35.9 (0, 71.9)
Processed meat (g·day^−1^)	12.0 (0, 29.7)
Poultry (g·day^−1^)	37.2 (9.6, 70.4)
Fish/seafood (g·day^−1^)	30.0 (3.6, 62.7)

Values represent mean ± standard deviation, median (25th, 75th percentiles), and the number of participants (%) ^1^ Data were collected at 0 weeks. ^2^ Data were collected at 8 weeks. ^3^ Data were collected at 26 weeks. BMI, body mass index; HbA_1c_, glycosylated hemoglobin A_1c_; HDL cholesterol, high-density lipoprotein cholesterol; HOMA-IR, homeostatic model assessment of insulin resistance; LDL cholesterol, low-density lipoprotein cholesterol.

## Data Availability

The dataset for this study is available from the corresponding author on reasonable request.

## Supplementary Materials

The following are available online at https://www.mdpi.com/article/10.3390/nu13113916/s1, Table S1: Calculations for cumulative average dietary intake, physical activity, and yearly changes in body weight and cardiometabolic risk factors, Table S2. Longitudinal associations of adherence to healthy plant-based diet with yearly changes in weight outcomes and cardiometabolic risk factors during weight-loss maintenance (n = 688), Table S3. Longitudinal associations of total grain and potato intake with yearly weight regain and changes in cardiometabolic risk factors during weight-loss maintenance (n = 710), Table S4. Longitudinal associations of legume intake with yearly weight regain and changes in cardiometabolic risk factors during weight-loss maintenance (n = 710), Table S5. Longitudinal associations of nut intake with yearly changes in waist circumference and cardiometabolic risk factors during weight-loss maintenance (n = 710), Table S6. Longitudinal associations of fruit intake with yearly weight regain and changes in cardiometabolic risk factors during weight-loss maintenance (n = 710), Table S7. Longitudinal associations of vegetable intake with yearly weight regain and changes in cardiometabolic risk factors during weight-loss maintenance (n = 710), Table S8. Longitudinal associations of vegetable and fruit intake with yearly weight regain and changes in cardiometabolic risk factors during weight-loss maintenance (n = 710), Figure S1. Changes in body weight during weight-loss maintenance in all participants (n = 688) (A) or completers (n = 493) (B) with lower or higher adherence to the plant-based diet.
