# Supplementary material for: Adherence to a Plant-Based Diet and Consumption of Specific Plant Foods—Associations with 3-Year Weight-Loss Maintenance and Cardiometabolic Risk Factors: A Secondary Analysis of the PREVIEW Intervention Study
Source: Nutrients. 2021 Nov 1;13(11):3916. doi: 10.3390/nu13113916 (PMC8618731; doi:10.3390/nu13113916)
Supplement: Supplementary file 1 [file nutrients-13-03916-s001.zip › nutrients-1417806-supplementary.pdf]

Supplementary Materials

Adherence to A Plant-based Diet and Consumption of Specific Plant Foods - Associations with 3-year Weight-loss Maintenance and Cardiometabolic Risk Factors: A Secondary Analysis of the PREVIEW Intervention Study

Statistical Analysis

Dietary diaries were not provided by participants at 8 weeks (the end of the weight loss phase) for 2 reasons. First, dietary instruction in each arm had not started at that time. Second, as participants were still being given food choice guidance e.g. daily eating plans and cooking books [1] at the start of the weight-loss maintenance phase (from 8 to 16 weeks), we hypothesized that dietary intake did not change that much during this period. By 26 weeks, we assumed their diets would be close to the target of each arm. Therefore, in the present study, 26-week diet and physical activity was used to estimate the average dietary intake and physical activity from 8 to 26 weeks.

References

1. Fogelholm, M.; Larsen, T.M.; Westerterp-Plantenga, M.; Macdonald, I.; Martinez, J.A.; Boyadjieva, N.; Poppitt, S.; Schlicht, W.; Stratton, G.; Sundvall, J., et al. PREVIEW: prevention of diabetes through lifestyle intervention and population studies in Europe and around the world. design, methods, and baseline participant description of an adult cohort enrolled into a three-year randomised clinical trial. *Nutrients* **2017**, *9*, 632, doi:10.3390/nu9060632.

Supplementary Materials

Adherence to A Plant-based Diet and Consumption of Specific Plant Foods - Associations with 3-year Weight-loss Maintenance and Cardiometabolic Risk Factors: A Secondary Analysis of the PREVIEW Intervention Study

Table S1. Calculations for cumulative average dietary intake, physical activity, and yearly changes in body weight and cardiometabolic risk factors

Intervals	Cumulative average dietary intake and physical activity	Yearly changes in body weight and cardiometabolic risk factors
8–26 weeks	Values at 26 weeks	Values at 26 weeks - values at 8 weeks
8–52 weeks	(Values at 26 weeks + values at 52 weeks)/2	Values at 52 weeks - values at 8 weeks
8–104 weeks	(Values at 26 weeks + values at 52 weeks + values at 104 weeks)/3	Values at 104 weeks - values at 8 weeks
8–156 weeks	(Values at 26 weeks + values at 52 weeks + values at 104 weeks + values at 156 weeks)/4	Values at 156 weeks - values at 8 weeks

Supplementary Materials

Adherence to A Plant-based Diet and Consumption of Specific Plant Foods - Associations with 3-year Weight-loss Maintenance and Cardiometabolic Risk Factors: A Secondary Analysis of the PREVIEW Intervention Study

Table S2. Longitudinal associations of adherence to healthy plant-based diet with yearly changes in weight outcomes and cardiometabolic risk factors during weight-loss maintenance (n=688)

Characteristic	Model 1[1]		Model 2[2]		Model 3[3]	
	Yearly mean change (95%CI)[4]	*P*-value	Yearly mean change (95%CI)	*P*-value	Yearly mean change (95%CI)	*P*-value
ΔFat mass (kg·year^{-1})	-0.17 (-0.38, 0.04)	0.106	-0.22 (-0.51, 0.07)	0.134	-	-
ΔWaist circumference (cm·year^{-1})	-0.13 (-0.35, 0.08)	0.231	-0.14 (-0.44, 0.16)	0.350	-	-
ΔFasting plasma glucose (mmol·L^{-1}·year^{-1})	-0.009 (-0.03, 0.01)	0.369	-0.03 (-0.06, 0.007)	0.123	-0.02 (-0.05, 0.01)	0.206
ΔHbA$_{1c}$ (mmol·mol^{-1}·year^{-1})	-0.05 (-0.13, 0.03)	0.257	-0.10 (-0.22, 0.01)	0.081	-0.07 (-0.18, 0.03)	0.179
ΔFasting insulin (mU·L^{-1}·year^{-1})	-0.0008 (-0.16, 0.16)	0.992	0.02 (-0.20, 0.24)	0.848	0.09 (-0.12, 0.31)	0.393
ΔHOMA-IR (year^{-1})	-0.007 (-0.006, 0.04)	0.774	-0.004 (-0.07, 0.06)	0.903	0.02 (-0.05, 0.08)	0.586
ΔSystolic blood pressure (mmHg·year^{-1})	-0.01 (-0.49, 0.47)	0.980	0.24 (-0.49, 0.97)	0.520	0.38 (-0.34, 1.09)	0.299
ΔDiastolic blood pressure (mmHg·year^{-1})	0.26 (-0.04, 0.55)	0.086	0.37 (-0.07, 0.82)	0.097	0.44 (0.0005, 0.87)	0.050
ΔTriacylglycerol (mmol·L^{-1}·year^{-1})	-0.01 (-0.02, 0.01)	0.476	-0.009 (-0.03, 0.02)	0.494	-0.002 (-0.03, 0.02)	0.845
ΔTotal cholesterol (mmol·L^{-1}·year^{-1})	-0.02 (-0.05, 0.01)	0.143	-0.03 (-0.07, 0.007)	0.109	-0.03 (-0.06, 0.01)	0.152
ΔHDL-cholesterol (mmol·L^{-1}·year^{-1})	0.003 (-0.003, 0.01)	0.311	0.009 (-0.0007, 0.02)	0.068	0.008 (-0.002, 0.02)	0.106

Analyses were performed using a linear mixed model with repeated measures. [1]Model 1 was adjusted for fixed factors including age, sex, ethnicity, BMI at 8 weeks, weight outcomes or cardiometabolic risk factors at 8 weeks, and time and random factors including study center and participant-ID.·[2]Model 2 was adjusted for confounders in model 1 plus fixed factors including time-varying physical activity, energy intake (kcal·day^{-1}), and alcohol intake (g·day^{-1}); for systolic blood pressure and diastolic blood pressure, model 2 was additionally adjusted for dietary sodium intake (g·day^{-1}). [3]Model 3 was adjusted for confounders in model 2 plus time-varying yearly weight change as a fixed factor. [4]Yearly mean change and 95% CI of main effects indicating changes in weight outcomes or cardiometabolic risk factors per year associated with 1-standard deviation increment in healthy plant-based diet index. HbA$_{1c}$, glycosylated hemoglobin A$_{1c}$; HDL-cholesterol, high-density lipoprotein cholesterol; HOMA-IR, homeostatic model assessment of insulin resistance.

Supplementary Materials

Adherence to A Plant-based Diet and Consumption of Specific Plant Foods - Associations with 3-year Weight-loss Maintenance and Cardiometabolic Risk Factors: A Secondary Analysis of the PREVIEW Intervention Study

Table S3. Longitudinal associations of total grain and potato intake with yearly weight regain and changes in cardiometabolic risk factors during weight-loss maintenance (*n*=710)

**Characteristic**	**Model 1**[1]		**Model 2**[2]		**Model 3**[3]	
	Yearly mean change (95%CI)[4]	*P*-value	Yearly mean change (95%CI)	*P*-value	Yearly mean change (95%CI)	*P*-value
ΔBody weight (kg·year^{-1})	0.05 (-0.04, 0.14)	0.246	0.13 (-0.02, 0.29)	0.084	-	-
ΔFat mass (kg·year^{-1})	0.01 (-0.09, 0.11)	0.842	0.04 (-0.13, 0.21)	0.626	-	-
ΔWaist circumference (cm·year^{-1})	0.02 (-0.08, 0.12)	0.706	0.13 (-0.06, 0.32)	0.174	-	-
ΔFasting plasma glucose (mmol·L^{-1}·year^{-1})	-0.0009 (-0.01, 0.01)	0.871	-0.003 (-0.03, 0.02)	0.768	-0.000003 (-0.02, 0.02)	0.996
ΔHbA$_{1c}$ (mmol·mol^{-1}·year^{-1})	0.01 (-0.03, 0.05)	0.512	0.02 (-0.05, 0.10)	0.521	0.02 (-0.05, 0.08)	0.657
ΔFasting insulin (mU·L^{-1}·year^{-1})	0.03 (-0.04, 0.11)	0.408	0.06 (-0.09, 0.20)	0.439	0.06 (-0.08, 0.19)	0.413
ΔHOMA-IR (year^{-1})	0.01 (-0.01, 0.04)	0.341	0.001 (-0.05, 0.05)	0.951	0.01 (-0.03, 0.06)	0.575
ΔSystolic blood pressure (mmHg·year^{-1})	0.29 (0.06, 0.52)	0.012	0.05 (-0.44, 0.54)	0.842	-0.02 (-0.93, 0.89)	0.966
ΔDiastolic blood pressure (mmHg·year^{-1})	0.09 (-0.04, 0.23)	0.189	-0.07 (-0.35, 0.22)	0.646	-0.08 (-0.36, 0.19)	0.552
ΔTriacylglycerol (mmol·L^{-1}·year^{-1})	0.007 (-0.003, 0.02)	0.156	0.01 (-0.009, 0.03)	0.293	0.008 (-0.009, 0.03)	0.349
ΔTotal cholesterol (mmol·L^{-1}·year^{-1})	0.00000005 (-0.06, 0.06)	1.000	-0.02 (-0.04, 0.005)	0.129	-0.02 (-0.04, 0.004)	0.111
ΔHDL-cholesterol (mmol·L^{-1}·year^{-1})	0.003 (-0.00004, 0.01)	0.053	-0.004 (-0.01, 0.002)	0.222	-0.003 (-0.009, 0.003)	0.308
ΔLDL-cholesterol (mmol·L^{-1}·year^{-1})	-0.003 (-0.01, 0.007)	0.562	-0.02 (-0.04, 0.001)	0.069	-0.02 (-0.04, 0.001)	0.064

Analyses were performed using a linear mixed model with repeated measures. [1]Model 1 was adjusted for fixed factors including age, sex, ethnicity, BMI at 8 weeks, body weight or cardiometabolic risk factors at 8 weeks, and time and random factors including study center and participant-ID. [2]Model 2 was adjusted for confounders in model 1 plus fixed factors including time-varying physical activity, energy intake (kJ·day^{-1}), alcohol intake (g·day^{-1}), animal-based food intake (g·day^{-1}), and other plant food intake (g·day^{-1}); for systolic blood pressure and diastolic blood pressure, model 2 was additionally adjusted for dietary sodium intake (g·day^{-1}). [3]Model 3 was adjusted confounders in model 2 plus time-varying yearly changes in body weight as a fixed factor. [4]Yearly mean change and 95% CI of main effects indicating changes in body weight or cardiometabolic risk factors per year associated with 75-g increment in total grains. HbA$_{1c}$, glycosylated hemoglobin A$_{1c}$; HDL-cholesterol, high-density lipoprotein cholesterol; HOMA-IR, homeostatic model assessment of insulin resistance; LDL-cholesterol, low-density lipoprotein cholesterol.

Supplementary Materials

Adherence to A Plant-based Diet and Consumption of Specific Plant Foods - Associations with 3-year Weight-loss Maintenance and Cardiometabolic Risk Factors: A Secondary Analysis of the PREVIEW Intervention Study

Table S4. Longitudinal associations of legume intake with yearly weight regain and changes in cardiometabolic risk factors during weight-loss maintenance (*n*=710)

Characteristic	Model 1[1]		Model 2[2]		Model 3[3]	
	Yearly mean change (95%CI)[4]	*P*-value	Yearly mean change (95%CI)	*P*-value	Yearly mean change (95%CI)	*P*-value
ΔBody weight (kg·year^{-1})	-0.01 (-0.04, 0.01)	0.318	-0.006 (-0.03, 0.02)	0.678	-	-
ΔFat mass (kg·year^{-1})	-0.03 (-0.06,-0.002)	0.034	-0.02 (-0.05, 0.01)	0.263	-	-
ΔWaist circumference (cm·year^{-1})	-0.03 (-0.06, -0.002)	0.037	-0.02 (-0.05, 0.02)	0.381	-	-
ΔFasting plasma glucose (mmol·L^{-1}·year^{-1})	-0.0002 (-0.003, 0.003)	0.920	0.0007 (-0.004, 0.005)	0.732	0.002 (-0.002, 0.006)	0.311
ΔHbA$_{1c}$ (mmol·mol^{-1}·year^{-1})	-0.004 (-0.01, 0.007)	0.447	-0.004 (-0.02, 0.009)	0.528	-0.001 (-0.014, 0.01)	0.844
ΔFasting insulin (mU·L^{-1}·year^{-1})	-0.01 (-0.03, 0.01)	0.276	-0.02 (-0.04, 0.01)	0.224	-0.008 (-0.03, 0.02)	0.556
ΔHOMA-IR (year^{-1})	-0.004 (-0.01, 0.004)	0.322	-0.005 (-0.01, 0.004)	0.284	-0.0008 (-0.009, 0.007)	0.844
ΔSystolic blood pressure (mmHg·year^{-1})	0.0008 (-0.07, 0.07)	0.983	0.07 (-0.01, 0.16)	0.102	0.08 (-0.09, 0.25)	0.372
ΔDiastolic blood pressure (mmHg·year^{-1})	0.02 (-0.02, 0.06)	0.401	0.03 (-0.02, 0.09)	0.178	0.04 (-0.007, 0.09)	0.096
ΔTriacylglycerol (mmol·L^{-1}·year^{-1})	-0.002 (-0.004, 0.0007)	0.157	-0.002 (-0.005, 0.001)	0.225	-0.001 (-0.004, 0.002)	0.402
ΔTotal cholesterol (mmol·L^{-1}·year^{-1})	-0.0001 (-0.004, 0.003)	0.953	-0.0002 (-0.005, 0.004)	0.923	0.0002 (-0.004, 0.004)	0.942
ΔLDL-cholesterol (mmol·L^{-1}·year^{-1})	-0.0003 (-0.003, 0.003)	0.854	-0.001 (-0.004, 0.003)	0.779	-0.0004 (-0.004, 0.003)	0.845

Analyses were performed using a linear mixed model with repeated measures. [1]Model 1 was adjusted for fixed factors including age, sex, ethnicity, BMI at 8 weeks, weight or cardiometabolic risk factors at 8 weeks, and time and random factors including study center and participant-ID. [2]Model 2 was adjusted for confounders in model 1 plus fixed factors including time-varying physical activity, energy intake (kJ·day^{-1}), alcohol intake (g·day^{-1}), animal-based food intake (g·day^{-1}), and other plant food intake (g·day^{-1}); for systolic blood pressure and diastolic blood pressure, model 2 was additionally adjusted for dietary sodium intake (g·day^{-1}). [3]Model 3 was adjusted confounders in model 2 plus time-varying yearly changes in body weight as a fixed factor. [4]Yearly mean change and 95% CI of main effects indicating changes in body weight or cardiometabolic risk factors per year associated with 10-g increment in legumes. HbA$_{1c}$, glycosylated hemoglobin A$_{1c}$; HOMA-IR, homeostatic model assessment of insulin resistance; LDL-cholesterol, low-density lipoprotein cholesterol.

Supplementary Materials

Adherence to A Plant-based Diet and Consumption of Specific Plant Foods - Associations with 3-year Weight-loss Maintenance and Cardiometabolic Risk Factors: A Secondary Analysis of the PREVIEW Intervention Study

Table S5. Longitudinal associations of nut intake with yearly changes in waist circumference and cardiometabolic risk factors during weight-loss maintenance (*n*=710)

Characteristic	Model 1[1]		Model 2[2]		Model 3[3]	
	Yearly mean change (95%CI)[4]	*P*-value	Yearly mean change (95%CI)	*P*-value	Yearly mean change (95%CI)	*P*-value
ΔWaist circumference ($cm·year^{-1}$)	-0.01 (-0.07, 0.04)	0.662	0.13 (-0.06, 0.32)	0.174	-	-
ΔFasting plasma glucose ($mmol·L^{-1}·year^{-1}$)	0.0007 (-0.005, 0.007)	0.871	-0.005 (-0.01, 0.004)	0.309	-0.002 (-0.01, 0.006)	0.599
ΔFasting insulin ($mU·L^{-1}·year^{-1}$)	0.02 (-0.02, 0.06)	0.271	-0.006 (-0.06, 0.05)	0.843	0.02 (-0.03, 0.07)	0.490
ΔHOMA-IR ($year^{-1}$)	0.004 (-0.005, 0.02)	0.552	-0.002 (-0.02, 0.02)	0.837	0.006 (-0.01, 0.02)	0.497
ΔSystolic blood pressure ($mmHg·year^{-1}$)	-0.07 (-0.20, 0.06)	0.288	-0.14 (-0.34, 0.03)	0.099	-0.20 (-0.58, 0.18)	0.304
ΔDiastolic blood pressure ($mmHg·year^{-1}$)	-0.05 (-0.12, 0.03)	0.220	-0.10 (-0.20, 0.009)	0.072	-0.08 (-0.18, 0.03)	0.141
ΔTriacylglycerol ($mmol·L^{-1}·year^{-1}$)	0.002 (-0.003, 0.007)	0.457	-0.01 (-0.01, 0.0005)	0.071	-0.004 (-0.01, 0.002)	0.231
ΔHDL-cholesterol ($mmol·L^{-1}·year^{-1}$)	-0.001 (-0.003, 0.0006)	0.229	-0.0009 (-0.003, 0.001)	0.409	-0.001 (-0.003, 0.0007)	0.202

Analyses were performed using a linear mixed model with repeated measures. [1]Model 1 was adjusted for fixed factors including age, sex, ethnicity, BMI at 8 weeks, waist circumference or cardiometabolic risk factors at 8 weeks, and time and random factors including study center and participant-ID. [2]Model 2 was adjusted for confounders in model 1 plus fixed factors including time-varying physical activity, energy intake ($kJ·day^{-1}$), alcohol intake ($g·day^{-1}$), animal-based food intake ($g·day^{-1}$), and other plant food intake ($g·day^{-1}$); for systolic blood pressure and diastolic blood pressure, model 2 was additionally adjusted for dietary sodium intake ($g·day^{-1}$). [3]Model 3 was adjusted confounders in model 2 plus time-varying yearly changes in body weight as a fixed factor [4]Yearly mean change and 95% CI of main effects indicating changes in waist circumference or cardiometabolic risk factors per year associated with 5-g increment in nuts. HDL-cholesterol, high-density lipoprotein cholesterol; HOMA-IR, homeostatic model assessment of insulin resistance.

Supplementary Materials

Adherence to A Plant-based Diet and Consumption of Specific Plant Foods - Associations with 3-year Weight-loss Maintenance and Cardiometabolic Risk Factors: A Secondary Analysis of the PREVIEW Intervention Study

Table S6. Longitudinal associations of fruit intake with yearly weight regain and changes in cardiometabolic risk factors during weight-loss maintenance (n=710)

Characteristic	Model 1[1]		Model 2[2]		Model 3[3]	
	Yearly mean change (95%CI)[4]	*P*-value	Yearly mean change (95%CI)	*P*-value	Yearly mean change (95%CI)	*P*-value
ΔBody weight (kg·year^{-1})	-0.01 (-0.07, 0.05)	0.744	-0.02 (-0.10, 0.05)	0.559	-	-
ΔFat mass (kg·year^{-1})	0.05 (-0.02, 0.12)	0.151	-0.008 (-0.09, 0.08)	0.857	-	-
ΔWaist circumference (cm·year^{-1})	-0.04 (-0.11, 0.03)	0.283	-0.03 (-0.12, 0.06)	0.550	-	-
ΔFasting plasma glucose (mmol·L^{-1}·year^{-1})	-0.005 (-0.01, 0.002)	0.184	-0.007 (-0.02, 0.004)	0.208	-0.005 (-0.02, 0.006)	0.351
ΔHbA$_{1c}$ (mmol·mol^{-1}·year^{-1})	-0.01 (-0.04, 0.01)	0.393	-0.02 (-0.06, 0.02)	0.260	-0.01 (-0.05, 0.02)	0.454
ΔFasting insulin (mU·L^{-1}·year^{-1})	-0.009 (-0.06, 0.04)	0.725	-0.02 (-0.09, 0.05)	0.526	-0.006 (-0.07, 0.06)	0.854
ΔHOMA-IR (year^{-1})	-0.006 (-0.02, 0.01)	0.491	-0.01 (-0.04, 0.01)	0.246	-0.006 (-0.03, 0.02)	0.569
ΔSystolic blood pressure (mmHg·year^{-1})	0.02 (-0.14, 0.17)	0.818	-0.14 (-0.37, 0.10)	0.257	-0.12 (-0.57, 0.33)	0.608
ΔTriacylglycerol (mmol·L^{-1}·year^{-1})	-0.003 (-0.009, 0.003)	0.402	0.004 (-0.005, 0.01)	0.348	0.005 (-0.003, 0.01)	0.200
ΔHDL-cholesterol (mmol·L^{-1}·year^{-1})	-0.0001 (-0.002, 0.002)	0.906	-0.001 (-0.004, 0.001)	0.336	-0.002 (-0.004, 0.001)	0.230

Analyses were performed using a linear mixed model with repeated measures. [1]Model 1 was adjusted for fixed factors including age, sex, ethnicity, BMI at 8 weeks, body weight or cardiometabolic risk factors at 8 weeks, and time and random factors including study center and participant-ID. [2]Model 2 was adjusted for confounders in model 1 plus fixed factors including time-varying physical activity, energy intake (kJ·day^{-1}), alcohol intake (g·day^{-1}), animal-based food intake (g·day^{-1}), and other plant food intake (g·day^{-1}); for systolic blood pressure, model 2 was additionally adjusted for dietary sodium intake (g·day^{-1}). [3]Model 3 was adjusted confounders in model 2 plus time-varying yearly changes in body weight as a fixed factor. [4]Yearly mean change and 95% CI of main effects indicating changes in body weight or cardiometabolic risk factors per year associated with 50-g increment in fruits. HbA$_{1c}$, glycosylated hemoglobin A$_{1c}$; HDL-cholesterol, high-density lipoprotein cholesterol; HOMA-IR, homeostatic model assessment of insulin resistance.

Supplementary Materials

Adherence to A Plant-based Diet and Consumption of Specific Plant Foods - Associations with 3-year Weight-loss Maintenance and Cardiometabolic Risk Factors: A Secondary Analysis of the PREVIEW Intervention Study

Table S7. Longitudinal associations of vegetable intake with yearly weight regain and changes in cardiometabolic risk factors during weight-loss maintenance (n=710)

Characteristic	Model 1[1]		Model 2[2]		Model 3[3]	
	Yearly mean change (95%CI)[4]	P-value	Yearly mean change (95%CI)	P-value	Yearly mean change (95%CI)	P-value
ΔBody weight (kg·year^{-1})	0.04 (-0.08, 0.16)	0.531	-0.09 (-0.24, 0.06)	0.227	-	-
ΔFat mass (kg·year^{-1})	-0.05 (-0.18, 0.08)	0.480	-0.06 (-0.22, 0.10)	0.432	-	-
ΔWaist circumference (cm·year^{-1})	0.01 (-0.13, 0.15)	0.860	-0.11 (-0.29, 0.06)	0.210	-	-
ΔFasting plasma glucose (mmol·L^{-1}·year^{-1})	0.006 (-0.009, 0.02)	0.408	0.003 (-0.03, 0.03)	0.767	0.009 (-0.01, 0.03)	0.374
ΔHbA_{1c} (mmol·mol^{-1}·year^{-1})	0.03 (-0.02, 0.08)	0.258	0.01 (-0.06, 0.08)	0.690	0.03 (-0.03, 0.09)	0.336
ΔFasting insulin (mU·L^{-1}·year^{-1})	0.02 (-0.09, 0.12)	0.733	0.01 (-0.12, 0.14)	0.880	0.05 (-0.07, 0.18)	0.408
ΔHOMA-IR (year^{-1})	0.005 (-0.03, 0.04)	0.777	0.001 (-0.04, 0.04)	0.958	0.02 (-0.02, 0.06)	0.308
ΔSystolic blood pressure (mmHg·year^{-1})	-0.08 (-0.40, 0.23)	0.602	-0.13 (-0.59, 0.32)	0.567	-0.37 (-1.23, 0.50)	0.406
ΔTotal cholesterol (mmol·L^{-1}·year^{-1})	0.01 (-0.004, 0.03)	0.133	0.008 (-0.01, 0.03)	0.439	0.01 (-0.01, 0.03)	0.369
ΔLDL-cholesterol (mmol·L^{-1}·year^{-1})	0.01 (-0.005, 0.02)	0.186	0.006 (-0.01, 0.02)	0.541	0.007 (-0.01, 0.03)	0.494

Analyses were performed using a linear mixed model with repeated measures.[1]Model 1 was adjusted for fixed factors including age, sex, ethnicity, BMI at 8 weeks, body weight or cardiometabolic risk factors at 8 weeks, and time and random factors including study center and participant-ID. [2]Model 2 was adjusted for confounders in model 1 plus fixed factors including time-varying physical activity, energy intake (kJ·day^{-1}), alcohol intake (g·day^{-1}), animal-based food intake (g·day^{-1}), and other plant food intake (g·day^{-1}); for systolic blood pressure, model 2 was additionally adjusted for dietary sodium intake (g·day^{-1}). [3]Model 3 was adjusted confounders in model 2 plus time-varying yearly changes in body weight as a fixed factor. [4]Yearly mean change and 95% CI of main effects indicating changes in body weight or cardiometabolic risk factors per year associated with 100-g increment in vegetables. HbA_{1c}, glycosylated hemoglobin A_{1c}; HOMA-IR, homeostatic model assessment of insulin resistance; LDL-cholesterol, low-density lipoprotein cholesterol.

Supplementary Materials

Adherence to A Plant-based Diet and Consumption of Specific Plant Foods - Associations with 3-year Weight-loss Maintenance and Cardiometabolic Risk Factors: A Secondary Analysis of the PREVIEW Intervention Study

Table S8. Longitudinal associations of vegetable and fruit intake with yearly weight regain and changes in cardiometabolic risk factors during weight-loss maintenance (*n*=710)

**Characteristic**	**Model 1**[1]		**Model 2**[2]		**Model 3**[3]	
	Yearly mean change (95%CI)[4]	*P*-value	Yearly mean change (95%CI)	*P*-value	Yearly mean change (95%CI)	*P*-value
ΔBody weight (kg·year^{-1})	0.01 (-0.11, 0.13)	0.857	-0.10 (-0.25, 0.05)	0.184	-	-
ΔFat mass (kg·year^{-1})	0.05 (-0.09, 0.18)	0.485	-0.06 (-0.22, 0.10)	0.467	-	-
ΔWaist circumference (cm·year^{-1})	-0.04 (-0.18, 0.09)	0.558	-0.13 (-0.31, 0.05)	0.163	-	-
ΔFasting plasma glucose (mmol·L^{-1}·year^{-1})	-0.002 (-0.02, 0.01)	0.735	-0.008 (-0.03, 0.01)	0.479	-0.0002 (-0.02, 0.02)	0.988
ΔHbA$_{1c}$ (mmol·mol^{-1}·year^{-1})	0.004 (-0.05, 0.05)	0.881	-0.02 (-0.09, 0.05)	0.592	0.006 (-0.06, 0.07)	0.850
ΔFasting insulin (mU·L^{-1}·year^{-1})	-0.001 (-0.10, 0.10)	0.979	-0.02 (-0.16, 0.11)	0.727	0.03 (-0.09, 0.16)	0.601
ΔHOMA-IR (year^{-1})	-0.005 (-0.04, 0.03)	0.781	-0.02 (-0.06, 0.03)	0.421	0.008 (-0.03, 0.05)	0.701
ΔTriacylglycerol (mmol·L^{-1}·year^{-1})	-0.01 (-0.03, -0.002)	0.021	-0.01 (-0.03, 0.005)	0.167	-0.007 (-0.02, 0.009)	0.399
ΔTotal cholesterol (mmol·L^{-1}·year^{-1})	-0.0001 (-0.02, 0.02)	0.990	-0.01 (-0.04, 0.008)	0.217	-0.01 (-0.03, 0.01)	0.292
ΔLDL-cholesterol (mmol·L^{-1}·year^{-1})	-0.00001 (-0.01, 0.01)	0.999	-0.02 (-0.03, 0.003)	0.108	-0.01 (-0.03, 0.004)	0.132

Analyses were performed using a linear mixed model with repeated measures.[1]Model 1 was adjusted for fixed factors including age, sex, ethnicity, BMI at 8 weeks, body weight or cardiometabolic risk factors at 8 weeks, and time and random factors including study center and participant-ID.[2]Model 2 was adjusted for confounders in model 1 plus fixed factors including time-varying physical activity, energy intake (kJ·day^{-1}), alcohol intake (g·day^{-1}), animal-based food intake (g·day^{-1}), and other plant food intake (g·day^{-1}); for systolic blood pressure and diastolic blood pressure, model 2 was additionally adjusted for dietary sodium intake (g·day^{-1}).[3]Model 3 was adjusted confounders in model 2 plus time-varying yearly changes in body weight as a fixed factor.[4]Yearly mean change and 95% CI of main effects indicating changes in body weight or cardiometabolic risk factors per year associated with 150-g increment in combined consumption of vegetables and fruits. HbA$_{1c}$, glycosylated hemoglobin A$_{1c}$; HOMA-IR, homeostatic model assessment of insulin resistance; LDL-cholesterol, low-density lipoprotein cholesterol.

Supplementary Materials

Adherence to A Plant-based Diet and Consumption of Specific Plant Foods - Associations with 3-year Weight-loss Maintenance and Cardiometabolic Risk Factors: A Secondary Analysis of the PREVIEW Intervention Study

A

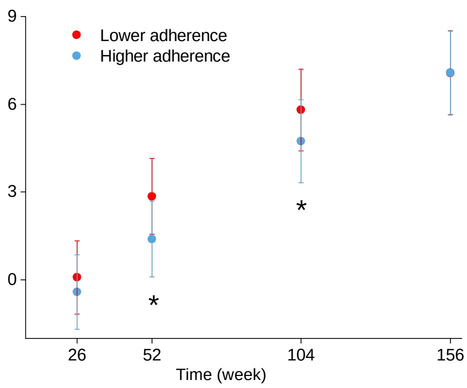

Time (week)		26	52	104	156
Lower adherence	Adjusted mean change in body weight from 8 weeks (kg)	0.1	2.9	5.8	7.1
	Lower 95%CI	-1.2	1.6	4.4	5.6
	Upper 95%CI	1.3	4.1	7.2	8.5
Higher adherence	Adjusted mean change in body weight from 8 weeks (kg)	-0.4	1.4	4.7	7.1
	Lower 95%CI	-1.7	0.1	3.3	5.7
	Upper 95%CI	0.9	2.7	6.1	8.5

B

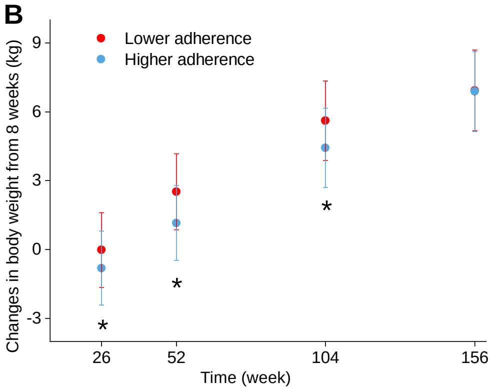

Time (week)		26	52	104	156
Lower adherence	Adjusted mean change in body weight from 8 weeks (kg)	-0.0	2.5	5.6	6.9
	Lower 95%CI	-1.6	0.9	3.9	5.2
	Upper 95%CI	1.6	4.2	7.3	8.7
Higher adherence	Adjusted mean change in body weight from 8 weeks (kg)	-0.8	1.2	4.4	6.9
	Lower 95%CI	-2.4	-0.5	2.7	5.1
	Upper 95%CI	0.8	2.8	6.2	8.6

Figure S1. Changes in body weight during weight-loss maintenance in all participants (n=688) (A) or completers (n=493) (B) with lower or higher adherence to the plant-based diet. The two groups were defined afresh according to the plant-based diet index at each time points. Values in the figure are adjusted means and 95% CI in changes in body weight. Analyses were performed using a linear mixed model with repeated measures, adjusted for age, sex, ethnicity, values of outcomes at 8 weeks, BMI at 8 weeks, time, physical activity, alcohol intake (g·day^{-1}), energy intake (kJ·day^{-1}) as fixed effects and participant-ID and study center as random effects. As the two groups were defined afresh at each time point, the two groups were compared at each time point, regardless of the significance of time and group interaction. *P<0.05.
